# Hyperhomocysteinemia exacerbates acute kidney injury *via* increased mitochondrial damage

**DOI:** 10.3389/fphys.2022.967104

**Published:** 2022-10-05

**Authors:** Mei Zhang, Rong Dong, Jingjing Da, Jing Yuan, Yan Zha, Yanjun Long

**Affiliations:** ^1^ Department of Nephrology, Guizhou Provincial Institute of Nephritic & Urinary Disease, Guizhou Provincial People’s Hospital, Guiyang, China; ^2^ Department of Biomedicine, Guizhou University School of Medicine, Guizhou University, Guiyang, China; ^3^ Department of Nephrology, People’s Hospital of Zhenfeng County, Qianxinan, Guizhou, China

**Keywords:** Hyperhomocysteinemia, acute kidney injury, mitochondrial damage, DNA damage, apoptosis

## Abstract

Acute kidney injury (AKI) is a complex and common set of multifactorial clinical syndromes, and associated with increased in-hospital mortality. There is increasing evidence that Hyperhomocysteinemia (HHcy) is highly associated with the development of a variety of kidney diseases, including AKI. However, the pathogenesis of HHcy in AKI remains unclear. In this study, we investigated the effect and mechanism of HHcy on cisplatin-induced AKI in mice and NRK-52E cells cultured with HHcy. We confirmed that mice with HHcy had higher serum levels of creatinine and more severe renal tubule injury after cisplatin injection. We found that HHcy aggravated renal mitochondrial damage, mainly manifested as decreased ATP β, significantly increased cytoplasmic Cyt C expression and the ADP/ATP ratio, and a significantly decreased mitochondrial DNA (mtDNA) copy number. In addition, we found that HHcy accelerated cisplatin-induced renal DNA damage; culturing NRK-52E cells with homocysteine (Hcy) could significantly increase apoptosis and mitochondrial damage. Interestingly, we found that Mdivi-1 reduced Hcy-induced mitochondrial damage, thereby reducing the level of apoptosis. In conclusion, these results suggest that HHcy might aggravate the development of AKI by increasing mitochondrial damage and that reducing Hcy levels or inhibiting mitochondrial damage may be a potential therapeutic strategy to delay the development of AKI.

## Introduction

Acute kidney injury (AKI) is a common clinical syndrome associated with high morbidity and in-hospital mortality. Globally, the proportion of hospitalized patients with AKI is between 0.7% and 31%; about 2 million people die from AKI each year (Hoste et al., 2015; Levey and James, 2017). The hospital fatality rate of AKI in China is 8.8%. In addition to the mortality rate, even if patients survive the acute disease, there will be the influence of chronic disease; AKI is very likely to develop into chronic kidney disease and accelerate the progression to end-stage renal disease (Wald et al., 2009; Heung and Chawla, 2012; Grams et al., 2016). Despite the increasing incidence of AKI, there is currently no effective treatment. However, it is important to actively seek and control risk factors for exacerbating AKI to improve the prognosis of patients with AKI.

Homocysteine (Hcy) is a thiol amino acid formed during the metabolic transformation of methionine to cysteine. Metabolic clearance of Hcy is mainly carried out by sulfur conversion and remethylation pathways. The normal level of Hcy in plasma is 5–15 μmol/L. When the body is deficient in important vitamin cofactors (folic acid, vitamin B6, vitamin B12) or enzymes (methylenetetrahydrofolate reductase, cystathionine-β-synthase, cystathionase), or the intake of methionine is excessive, the concentration of Hcy in serum or plasma will increase abnormally (Hannibal and Blom, 2017; Sbodio et al., 2019; Portillo et al., 2020).

Hyperhomocysteinemia (HHcy) results in an abnormal increase in plasma Hcy concentration (>15 μmol/L), and can be divided into three stages, according to plasma Hcy concentration: mild (15–30 μmol/L), moderate (31–100 μmol/L), and severe (>100 μmol/L) (A. Kumar et al., 2017). According to a survey (Yang et al., 2015), the prevalence of HHcy has been increasing in China in recent years, and is about 27.5%, especially in the north. Studies have shown that HHcy is associated with the pathophysiology of glomerulosclerosis and interstitial fibrosis, leading to progressive decline in renal function (Pushpakumar et al., 2019). Mild and moderate HHcy are prevalent in patients with chronic kidney disease; HHcy has been recognized as an independent risk factor for chronic kidney disease (C. H. Chen et al., 2016; Kong et al., 2017; Ji et al., 2019).

In recent years, the role and potential mechanism of HHcy in AKI have also attracted the attention of many researchers (Long and Nie, 2016; Abate et al., 2020; Zhang et al., 2020). On analyzing serum samples from 17 hospitalized patients with AKI, Sun et al. (2012) found significantly higher concentrations of Hcy in the serum of patients with AKI than in that of subjects with normal renal function. We recently reported that HHcy might aggravate the development of AKI in mice through endoplasmic reticulum stress, oxidative stress, and DNA damage. In addition, Li et al. (2019) found that increased Hcy levels in mice would exacerbate AKI.

Mitochondria are highly dynamic organelles that undergo a coordinated cycle of division and fusion to maintain their shape, distribution, and size. These balanced, dynamic changes not only affect the mitochondrial quality control, but also are extremely important in cell cycle, immunity, and apoptosis (Van Der Bliek et al., 2017). It has been reported that AKI caused by sepsis, ischemia, or renal toxicity involves changes in mitochondrial injury in renal tubular epithelial cells, and especially mitochondrial fracture in the proximal tubules, which are extremely rich in mitochondrial content (Ishimoto and Inagi, 2016; Szeto, 2017; Sun et al., 2019). In recent years, more and more studies have shown that HHcy may accelerate cell apoptosis by mediating mitochondrial damage (Sipkens et al., 2012; Kumar et al., 2018). Cueto et al. (2018) hypothesized that HHcy induces mitochondrial injury by inhibiting gene expression of mitochondrial complex I. In addition, Chen et al. (2017) showed that Hcy could induce mitochondrial damage and promote the development of cerebral infarction. Therefore, we speculate that HHcy might mediate the development of AKI aggravated by mitochondrial injury.

In this study, we explored the role and potential mechanism of HHcy in the development of AKI by using a clinically relevant mouse model of cisplatin-induced AKI and an *in vitro* model of intervention, using NRK-52E cells. We confirmed that HHcy can promote the development of AKI in mice, and that the possible underlying mechanism is the increase of Hcy level aggravating the mitochondrial damage pathway. Mitochondrial injury increases the apoptosis of renal tubular epithelial cells. Thus, we deduce that limiting HHcy-mediated mitochondrial damage may reduce kidney damage.

## Materials and methods

### Animals

Forty-two male C57BL/6 mice (6–8 weeks, 19 ± 2 g) were purchased from Liaoning Changsheng Biotechnology Co., Ltd (Liaoning, China). All mice were fed in a temperature- and humidity-controlled room (temperature, 22°C ± 2°C; humidity, 55% ± 2%) with a 12 h–12 h light-dark cycle. Water and food were provided *ad libitum*. All studies were approved by the ethics committee of the Guizhou Provincial People’s Hospital [Approval number: (2019)070] and the study protocol was approved by the institutional animal care and welfare committee.

### Animal models

To establish the HHcy model, all mice were randomly assigned to two groups: one group was fed a regular diet of standard rodent chow and the other group (the H-Met group) was fed a diet rich in methionine [19.56 g/kg (2%) methionine] for 3 weeks. AKI was established by intraperitoneal injection of cisplatin (P4393; Sigma-Aldrich; 24 mg/kg body weight) (Long et al., 2017). All mice were humanely euthanised at day 1 or day 3 after cisplatin injection, and blood and kidney samples were harvested.

### Cell culture and treatment

Normal rat kidney epithelial cells (cell strain NRK-52E, *Rattus norvegicus*) were cultured in F12 medium supplemented with 10% fetal bovine serum (Gibco, Carlsbad, CA, United States) in an incubator at 37°C, 5% CO_2_. To investigate whether Hcy aggravates mitochondrial damage *in vitro*, cells were treated with DL-homocysteine (H4628, Sigma-Aldrich, United States) for four different periods (0 h, 4 h, 8 h, 16 h) after approximately 60% confluence was reached (Long et al., 2017). The expression of mitochondrial markers (Cyt C and ATP β) was detected using western blotting. To inhibit mitochondrial damage, cells were pretreated with 10 µM Mdivi-1 (S7162, Selleck, United States) for 1 h before DL-homocysteine stimulation (Liu et al., 2019), and the experiments were repeated three times. Subsequently, whole cell lysates were collected for further analysis.

### Serum Hcy measurement

Serum Hcy was measured using a homocysteine assay kit (Ausa, China) and an automatic clinical analyzer (Beckman Coulter, CA, United States) according to the manufacturer’s instructions. The Hcy measurement was standardized according to the National Institute of Standards and Technology Standard Reference Material (SRM 1955).

### Biochemical analysis

Blood samples were evaluated by measuring creatinine using a blood creatinine assay kit (DICT-500, Bioassay Systems, United States), according to the manufacturer’s instructions.

### Histology

For histological analysis, the paraffin-embedded kidney was sliced into 2-µm sections, which were subjected to hematoxylin and eosin (H&E) staining (G1120, Solarbio, China), or periodic acid–Schiff staining (G1281, Solarbio, China) according to the manufacturer’s instructions. Tubule injury scores were determined by grading tubular dilatation, tubular casting formation, cell necrosis, and brush edge loss. At least 10 fields viewed using a microscope (×400) were randomly selected and scored as follows: 0, no damage; 1, less than 25%; 2, 25%–50%; 3, 50%–75%; 4, >75% (Zhang et al., 2020).

### TUNEL staining

Renal tissue sections (4 µm) were stained using an *in-situ* cell death detection kit (C1088; Beyotime, China) to detect the viability of tubular cell. The number of TUNEL-positive cells was calculated in 10 randomly selected fields per section under fluorescence microscopy (×400).

### ADP/ATP ratio measurement

The ADP/ATP ratio in kidney tissue was measured with an EnzyLight™ ADP/ATP ratio assay Kit (ELDT-100, BioAssay United States), according to manufacturer’s instructions.

### Transmission electron microscopy

To assess mitochondrial morphological changes, fresh kidney tissue was fixed with 2.5% glutaraldehyde, dehydrated, and embedded with EPON resin. Ultrathin sections were stained with 2% uranyl acetate and 2.6% lead citrate solution for observation using transmission electron microscopy (HT7800; Hitachi, Tokyo, Japan). As previously described, mitochondrial damage was scored on a grade of 0–4 on the basis of the typical ultrastructural features of mitochondria: 0, complete mitochondrial structure; 1, early mitochondrial swelling and cristae separation; 2, more marked swelling and cristae separation, more extensive mitochondrial swelling and rupture of cristae; 4, severe mitochondrial swelling, rupture of cristae and disruption of mitochondrial membranes. At least ten fields of per kidney randomly selected were evaluated in a blinded manner (Chen et al., 2017).

### Flow cytometry analysis

After treatment, NRK-52E cells were washed with precooled phosphate buffered saline and costained with Annexin V-APC and propidium iodide (MultiSciences Biotech Co., Ltd). Apoptosis was evaluated using a BD FACSCalibur flow cytometer.

### Immunohistochemical staining

After 4-µm kidney sections were deparaffinized and hydrated, each section was exposed to 3% hydrogen peroxide solution. After antigen repair, kidney sections were incubated with primary antibodies against γ H2AX (1:200, Abcam, ab2893) overnight at 4°C. Subsequently, sections were incubated with HRP-conjugated secondary antibody (Proteintech Group, Inc., Chicago, IL, United States) at room temperature for 30 min; a DAB kit (Zhongshan Jinqiao Biotechnology Co., Ltd., Beijing, China) was used for localization. Images were taken through a microscope (×400).

### Preparation of cytoplasmic and mitochondrial fractions

Mitochondrial and cytoplasmic proteins were collected using tissue or cell mitochondrial isolation kits (C3606; Beyotime, China), according to the manufacturer’s instructions. Briefly, renal tissues or cells were washed with precooled phosphate buffered saline, and lysis buffer was added. After dissolving the mixture on ice for 30 min, the lysates were centrifuged at ×5000*g* for 15 min at 4°C; the resulting precipitate was mitochondrial protein. Further, the cytoplasmic protein was isolated from the supernatant by centrifuging at ×12000*g* for 10 min at 4°C.

### Western blotting

Protein samples for western blotting were obtained from kidney tissue with precooled RIPA buffer (Beyotime, Haimen, China) containing a protease inhibitor cocktail. The protein samples were boiled with SDS loading buffer after the protein concentration was measured using a BCA protein assay kit (PC0020; Solarbio, China). A total of 25 µg of protein was separated by 10%–15% SDS-PAGE and transferred to PVDF membranes. The membranes were blocked using non-fat milk for 2 h at room temperature. Membranes were subsequently probed overnight at 4°C with primary antibodies against ATP β (1:2000, Abcam, ab14730), caspase-3 (1:2000, Abcam, ab184787), caspase-12 (1:2000, Abcam, ab62463), cleaved caspase-3 (1:1000, Cell Signaling, 9664T), Cyt C (1:3000, Abcam, ab133504), GAPDH (1:2000, Cell Signaling, 14C10), β-actin (1:1000, Abcam, ab8227), γ H2AX (1:1000, Abcam, ab2893). After washing with 1× TBST buffer, HRP-conjugated secondary antibody was incubated at 37°C for 2 h. The protein bands on western blots were then detected using ECL plus (WBKLS0100, Millipore, United States), according to the manufacturer’s instructions.

### Mitochondrial DNA copy number assay

The relative mtDNA copy number was measured by calculating the ratio of mtDNA to nuclear DNA, as described previously. Briefly, total DNA was extracted from frozen kidney tissues or cells using the universal Genomic DNA Kit (Takara, Japan) according to the manufacturer’s instructions. Real-time PCR was performed using a Talent qPCR PreMix (SYBR Green) (TIANGEN, Beijing, China) in a real-time PCR apparatus (Bio-Rad, CA, United States), and 20 ng of the DNA was used for qPCR analysis. The mtDNA levels were determined by comparing the cycle threshold (Ct) of mitochondrial genes (ND1) and nuclear genes (β-actin). The conditions for qPCR were: 95°C for 3 min followed by 40 cycles of 95°C for 15 s and 60°C for 1 min. Relative expression of mtDNA was calculated using the 2^−ΔΔCt^ method. The sequences of the primers are given in Table 1 (Ylikallio et al., 2010; Tsuji et al., 2016; Cioffi et al., 2017; Hu et al., 2018).

**TABLE 1:** Sequence of primers used for RT-PCR.

Species	Gene	Sequence (5′ → 3′)	Accession no.
Mouse	*ND1*	F: GGA​TCC​GAG​CAT​CTT​ATC​CA	AY394056.1
		R: GGT​GGT​ACT​CCC​GCT​GTA​AA	
Mouse	*β-actin*	F: GGA​AAA​GAG​CCT​CAG​GGC​AT	NM_007393
		R: GAA​GAG​CTA​TGA​GCT​GCC​TGA	
Rat	*ND1*	F: CTA​CGC​AAA​GGC​CCC​AAC​AT	EU104717.1
		R: TAG​AGC​TAG​TGT​AAG​GGA​GAG​GG	
Rat	*β-actin*	F: CTG​CTC​TTT​CCC​AGA​TGA​GG	V01217.1
		R: CCA​CAG​CAC​TGT​AGG​GGT​TT	

### Statistical analysis

Data were expressed as mean ± standard deviation (SD); Statistical analysis was performed by Shapiro–Wilk normality, Kolmogorov–Smirnov normality using GraphPad Prism version 5 (GraphPad Software, La Jolla, CA, United States). For comparisons between groups with normally distributed data were analyzed using the *t-test* and one-way analysis of variance (ANOVA). The Mann-Whitney and Kruskal–Wallis non-parametric tests were used for non-normally distributed variables. Significance was assumed for *p* < 0.05.

## Results

### HHcy accelerates cisplatin-induced DNA damage in mice

**FIGURE 1:**
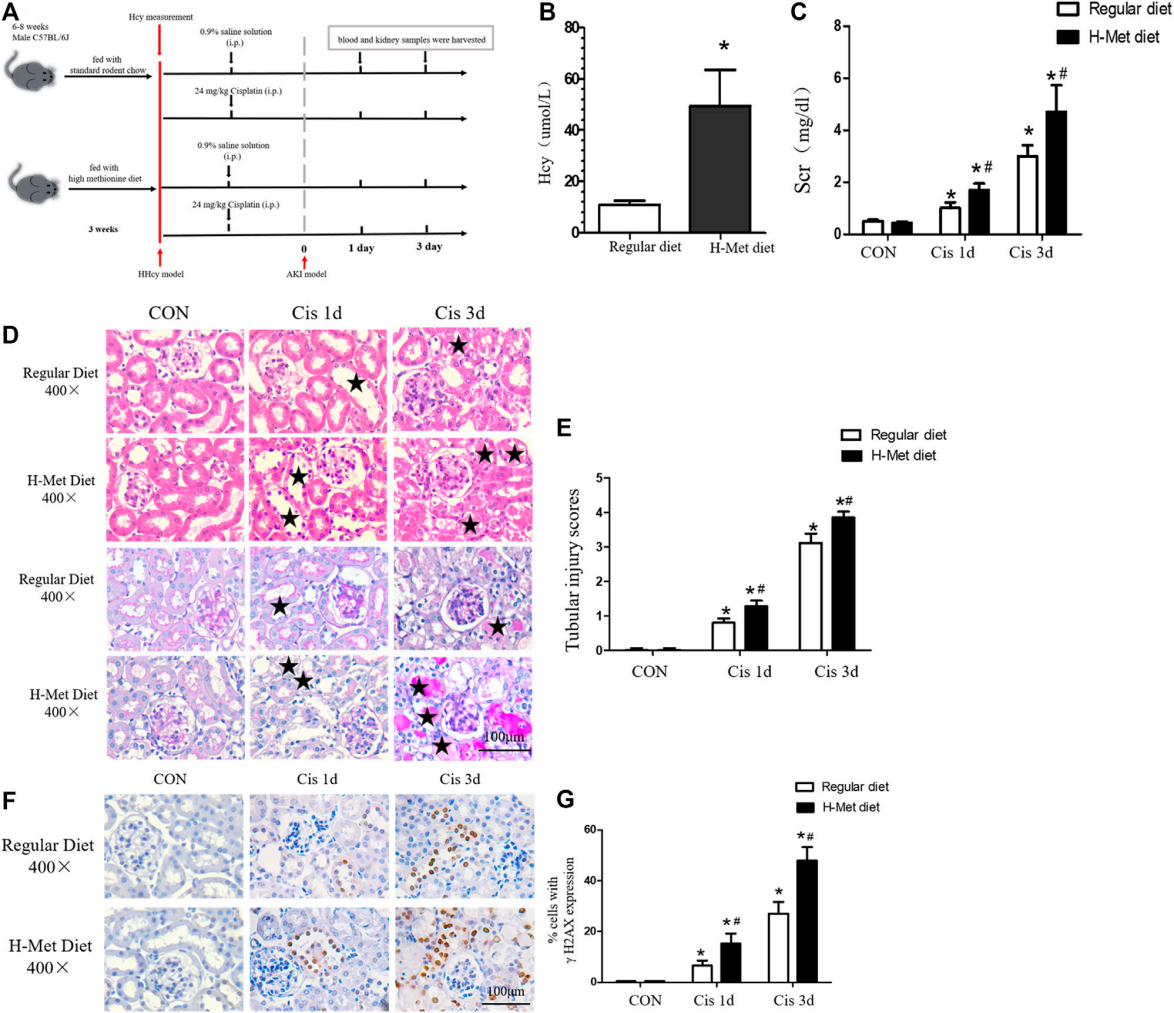
Mice with HHcy are more susceptible to cisplatin-induced acute kidney injury. **(A)** Timeline of mouse experiment. **(B)** Plasma level of homocysteine (Hcy) in mice. **(C)** Level of serum creatinine (Scr) at days 1 and 3 after cisplatin injection. **(D)** Representative kidney histology of H&E staining and periodic acid–Schiff staining at days 1 and 3 after cisplatin injection (scale bar, 100 μm; magnification, ×400). Black stars (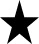) indicate tubular damage in renal tissue, including tubular dilatation, tubular casting formation, cell necrosis, and brush edge loss. **(E)** Statistical quantification analysis showing injury score of H&E staining. **(F)** Representative images of immunohistochemical staining show γ H2AX-positive kidney cells in at days 1 and 3 after cisplatin injection (scale bar, 100 μm; magnification ×400). **(G)** Quantitative determination of γ H2AX-positive cells. Data expressed as mean ± SD; *n* = 5–7 per group. ^*^ *p* < 0.05 versus control; ^#^ *p* < 0.05 versus mice fed with regular diet.

Our previous studies have shown that HHcy can aggravate AKI induced by ischemia-reperfusion or cisplatin (Zhang et al., 2021). However, the specific pathogenesis is still unclear. The mitochondrial damage pathway plays an important role in AKI, and we speculated whether HHcy could promote the development of AKI by aggravating mitochondrial damage. We established mouse models of HHcy and AKI, and collected blood and kidney samples (Figure 1A). After feeding C57BL/6J mice with a high-methionine diet (19.56 g/kg methionine) for 3 weeks, we found that the plasma level of Hcy in mice fed a high-methionine (H-Met) diet was significantly higher than in mice fed a regular diet (10.90 ± 1.62 µM vs. 49.56 ± 14.08 µM) (Figure 1B). After a single intraperitoneal injection of 24 mg/kg cisplatin for 24 or 72 h, serum creatinine (Scr) levels and renal tubular injury scores were significantly increased in the H-Met group compared with the group fed a regular diet (Figures 1C–E), but there were no significant differences in Scr levels and renal tubule injury scores between the two groups of mice without cisplatin treatment (Figures 1C–E). These results suggest that HHcy can promote kidney injury induced by cisplatin in mice.

Because DNA damage can activate mitochondrial damage pathways (Saki and Prakash, 2017), we examined the expression of DNA damage markers (γ H2AX) in the kidneys by immunohistochemical analysis after cisplatin injection. As shown in Figures 1F,G, after 1 or 3 days of cisplatin injection, the expression of γ H2AX in renal tubules in HHcy mice was significantly greater than that in control mice. These results suggest that HHcy might accelerate the activation of the mitochondrial injury signaling pathway.

### HHcy promotes mitochondrial damage in kidney after cisplatin injection

**FIGURE 2:**
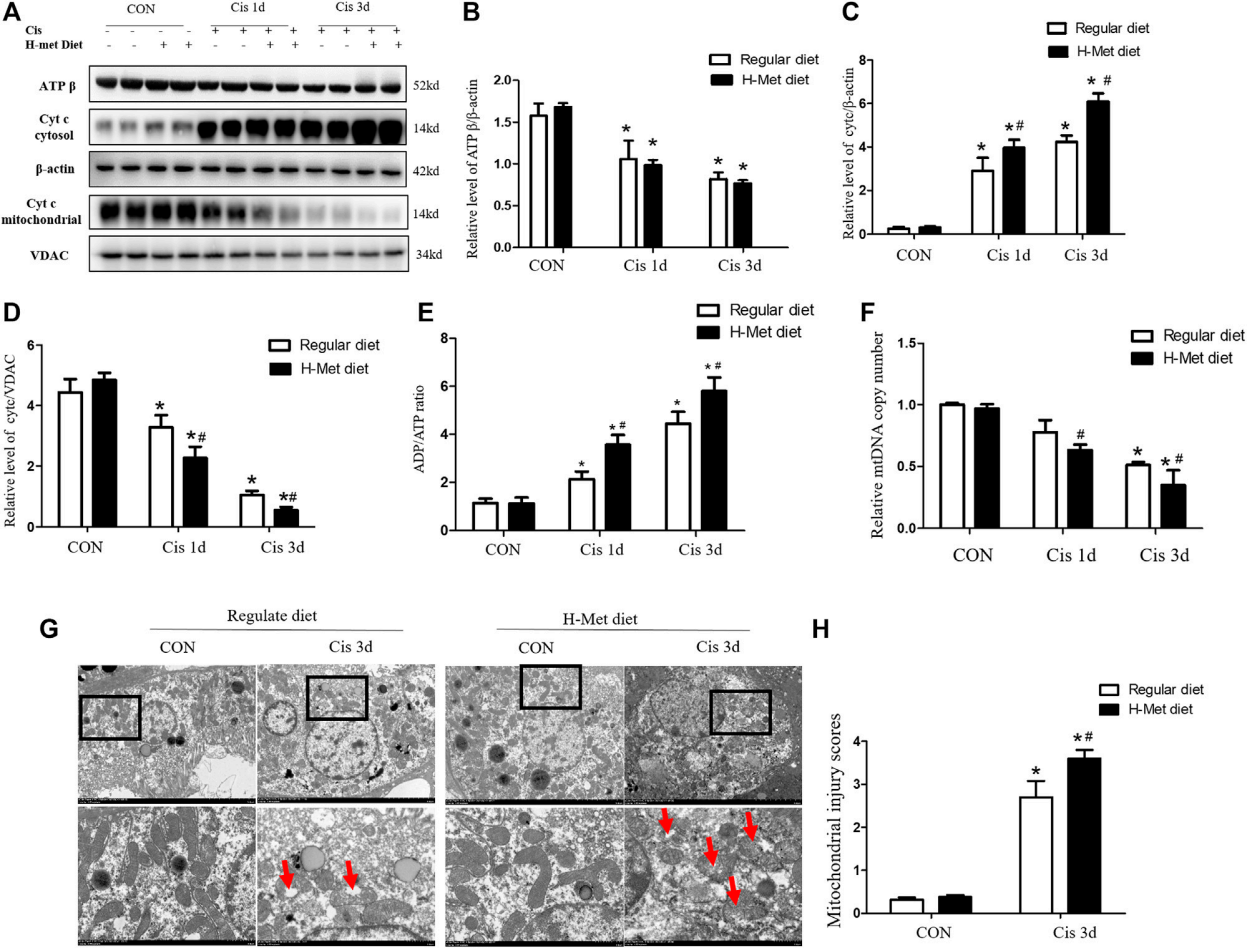
Hyperhomocysteinemia promotes cisplatin-induced mitochondrial damage of kidney in mice. **(A**–**D)** Western blot analysis of ATP β and Cyt C (in cytoplasm or mitochondria) in kidney after cisplatin injection. **(E)** Level of the ATP/ADP ratio in mice. **(F)** Quantification of mtDNA copy number. **(G)** Electron micrographs reveal obvious morphological changes of mitochondria in renal tissue of HHcy mice at day 3 after cisplatin injection. Upper panel: scale bar, 5 μm; magnification 3000×. Lower panel: scale bar, 1 μm; magnification 8000×. The red arrows (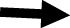)suggest changes in mitochondrial structure in the proximal renal tubular cells, including mitochondrial shortening and roundness, mitochondrial fragmentation, mitochondrial swelling and vacuoles, crest loss. **(H)** Statistical quantification analysis showing injury score of mitochondrial. Data expressed as mean ± SD; *n* = 5–7 per group. ^*^ *p* < 0.05 versus control; ^#^ *p* < 0.05 versus mice fed with regular diet.

To further explore the role of HHcy in mitochondrial injury, we determined the levels of Cyt C and ATP β expressed by proteins related to renal mitochondrial injury by western blotting. Levels of ATP β were significantly reduced in mice treated with cisplatin compared with control mice receiving the same diet. After cisplatin, the ATP β level of mice in the H-Met group was slightly lower than that in the regular diet group, but the difference was not statistically significant (Figures 2A,B). However, the level of ADP/ATP ratio meaningfully increased in the H-Met group compared to the regular diet group after cisplatin (Figure 2E). After 1 or 3 days of cisplatin treatment, HHcy accelerated Cyt C release to cytoplasm compared with mice in the regular diet group (Figures 2A,C,D). After cisplatin injection, the mtDNA copy number of mice in the H-Met diet group was significantly smaller than that in the regular diet group (Figure 2F). In addition, we observed changes in mitochondrial structure in proximal renal tubules using electron microscopy. As shown in Figures 2G,H, mitochondria of mouse proximal tubular cells in the control group showed a typical filamentous morphology. While proximal renal tubular cells of HHcy mice were shortened or spherical, with significant structural changes (mitochondrial fragmentation, mitochondrial swelling, vacuole, cristae fracture), after cisplatin injection. Taken together, these results suggest that mitochondrial damage plays an important role in HHcy exacerbation of cisplatin-induced AKI.

### HHcy mice exhibits increased tubular cell apoptosis after cisplatin injection

**FIGURE 3:**
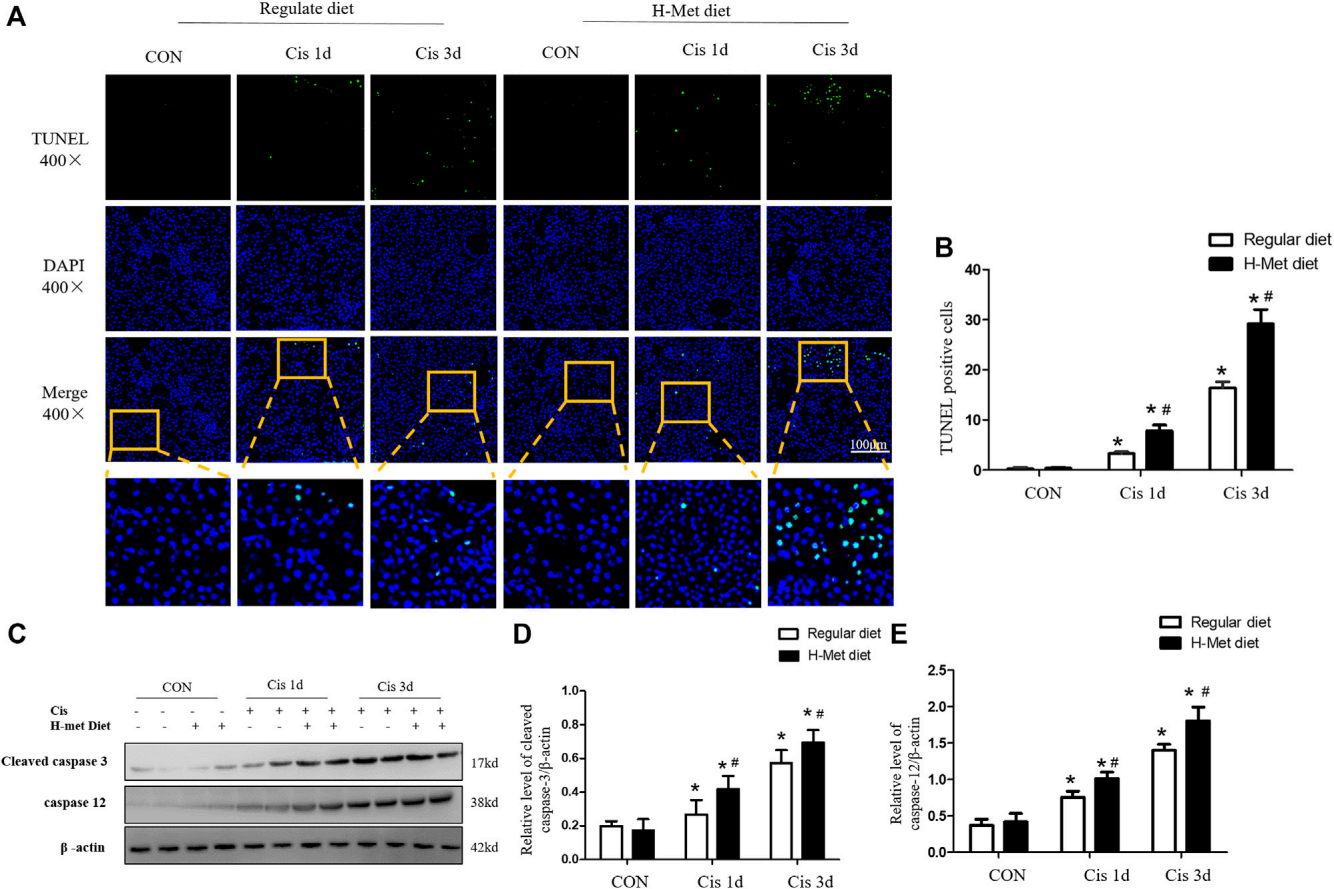
Hyperhomocysteinemia aggravates cisplatin-induced renal tubular epithelial cell apoptosis in mice. **(A)** Representative micrographs show the viability of tubular cell detected by TUNEL staining after cisplatin injection at days 1 and 3. **(B)** Quantitative determination of TUNEL-positive tubular cells after cisplatin injection (scale bar, 100 μm; In review magnification, ×400). **(C**–**E)** Western blot analysis of protein level of cleaved caspase-3 and caspase 12 in kidney after cisplatin injection. Data are expressed as mean ± SD, *n* = 5–7 per group. ^*^ *p* < 0.05 versus control; ^#^ *p* < 0.05 versus mice fed with regular diet.

The mitochondrial injury pathway is one of the main pathways of renal tubular epithelial cells. The decrease in mtDNA copy number and the release of Cyt C from mitochondria to cytosol are markers of the internal pathways of apoptosis (Bhargava and Schnellmann, 2017; Cheng et al., 2020). We further evaluated the viability of tubular cell in the renal cortex using a TUNEL assay. As shown in Figures 3A,B, the number of TUNEL-positive tubular cells in mice injected with cisplatin for 1 or 3 days was significantly larger than that in the control group, whereas the H-Met diet significantly increased the number of TUNEL-positive tubular cells after cisplatin injection. Then, the expression of apoptosis-related proteins (cleaved caspase-3 and caspase-12) was detected by Western blotting, as shown in Figures 3C–E, compared with the regular diet group, cleaved caspase-3 and caspase-12 were significantly increased in mice fed H-Met diet after cisplatin injection. These results showed that HHcy decreased the viability of tubular cells and aggravated cell apoptosis after cisplatin injection.

### HHcy causes tubular cell apoptosis by aggravating mitochondrial damage *in vitro*

**FIGURE 4:**
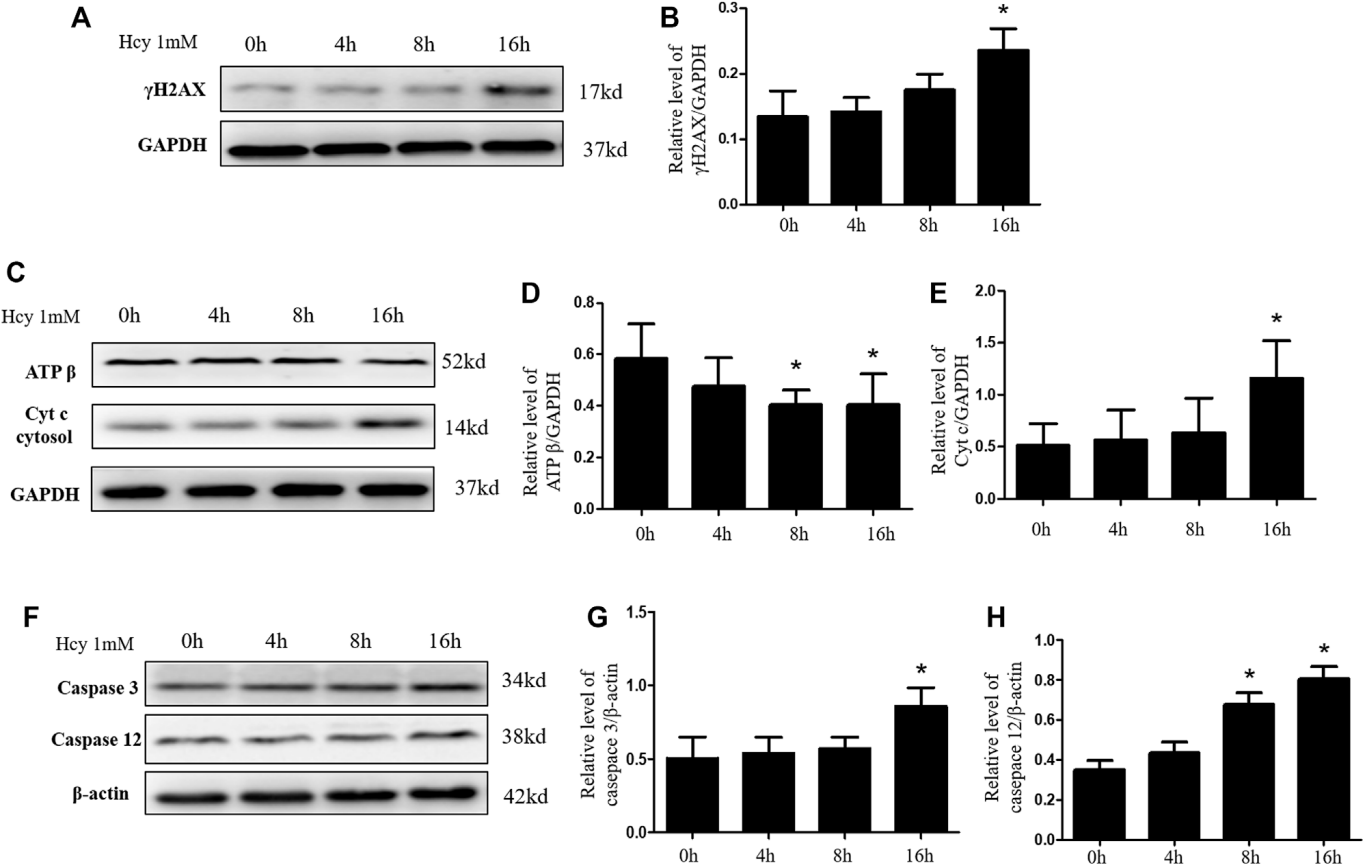
Hyperhomocysteinemia aggravates DNA damage and mitochondrial damage *in vitro*. **(A**,**C**,**F)** Representative western blots show the levels of γ H2AX, ATP β, Cyt C, Caspase 3, and Caspase 12 in NRK-52E cells after homocysteine stimulation. **(B**,**D**,**E**,**G**,**H)** Relative levels of: **(B)** γ H2AX, **(D)** ATP β, **(E)** Cyt C, **(G)** caspase 3, and **(H)** caspase 12. Data expressed as mean ± SD, *n* = 3 per group. ^*^ *p* < 0.05 versus 0 h.

The increased apoptosis of renal tubular cell in HHcy mice after cisplatin may be due to HHcy-induced mitochondrial damage. To confirm whether HHcy directly affects mitochondria, we treated rat proximal tubular cells (NRK-52E) with 1 mM homocysteine for 0, 4, 8, or 16 h. Similar to *in vivo* results, western blot analysis demonstrated that, compared with the control group, the protein expression of γ H2AX increased significantly after 1 mM homocysteine stimulation for 16 h (Figures 4A,B). In addition, after stimulation of NRK-52E cells with 1 mM homocysteine, the protein expression of ATP β was significantly decreased at 8 h and 16 h (Figures 4C,D), while the protein expression of Cyt C in cytoplasm was significantly increased at 16 h (Figures 4C,E). Western blotting was used to further detect the protein expression of caspase 3 and caspase 12 at different periods. As shown in Figures 4F–H, the expressions of caspase 3 and caspase 12 were significantly increased at 16 h after Hcy stimulation compared with the control group. These results are consistent with those obtained in the *in vivo* experiments, suggesting that HHcy may directly mediate mitochondrial damage and eventually lead to cell apoptosis *in vitro*.

### Mdivi-1 attenuates HHcy-induced mitochondrial damage and apoptosis

**FIGURE 5:**
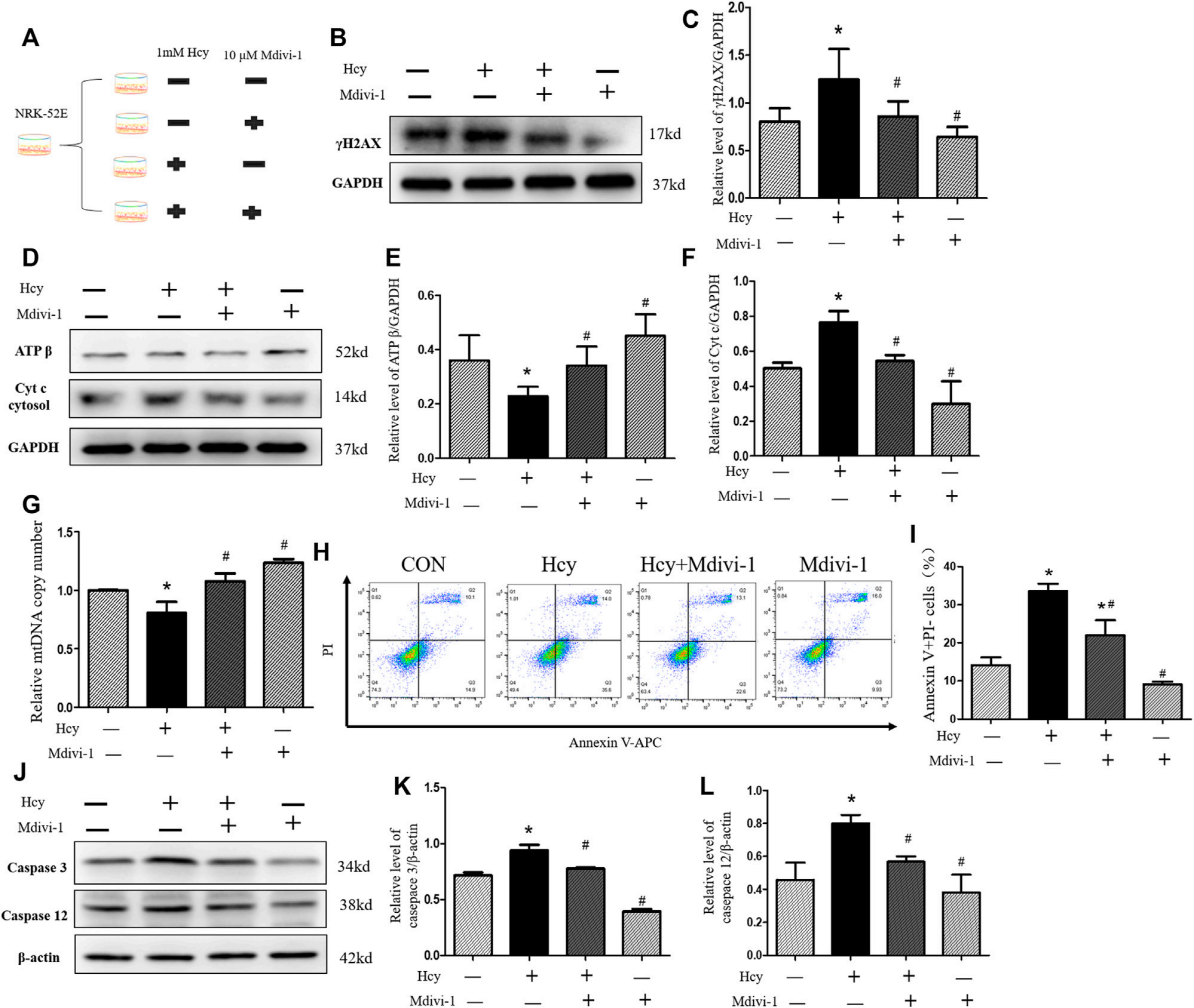
Amelioration by Mdivi-1 of HHcy-induced mitochondrial damage *in vitro*. **(A)** Mdivi-1 intervention: NRK-52E cells were treated with 1 mM of homocysteine with or without 10 µM Mdivi-1 for 16 h **(B**,**D)** Representative western blots show the levels of γ H2AX, ATP β, and Cyt C after Mdivi-1 intervention in NRK-52E cells. **(C**,**E**,**F)** Relative levels of: **(C)** γ H2AX; **(E)** ATP β; and **(F)** Cyt C after Mdivi-1 intervention in NRK-52E cells. **(G)** Real-time PCR shows mtDNA copy number. **(H)** Flow cytometry assays show cell apoptosis in NRK-52E cells after Mdivi-1 intervention in NRK-52E cells. **(I)** Percentage of apoptotic cells. **(J)** Representative western blots show the levels of caspase 3 and caspase 12 after Mdivi-1 intervention in NRK-52E cells. **(K**,**L)** Relative levels of: **(K)** caspase 3; **(L)** caspase 12 after Mdivi-1 intervention in NRK-52E cells. Data expressed as mean ± SD, *n* = 3 per group. ^*^ *p* < 0.05 versus control group; ^#^ *p* < 0.05 versus cells treated with homocysteine.

We next investigated whether Mdivi-1 can alleviate HHcy-induced mitochondrial damage and apoptosis. Because mitochondrial damage was significantly increased 16 h after Hcy treatment, NRK-52E cells were further incubated with 1 mM homocysteine and 10 µM Mdivi-1, an inhibitor of mitochondrial division (Figure 5A). Interestingly, compared with the Hcy group, the expression of γ H2AX protein was significantly decreased after Mdivi-1 intervention (Figures 5B,C). As shown in Figures 5D–F, compared with the Hcy group, the release rate of Cyt C into the cytoplasm was significantly improved in the Hcy + Mdivi-1 group, and the expression level of ATP β was significantly increased. Similarly, qPCR results showed that mtDNA copy number increased significantly after mdivi-1 stimulation (Figure 5G).

**FIGURE 6:**
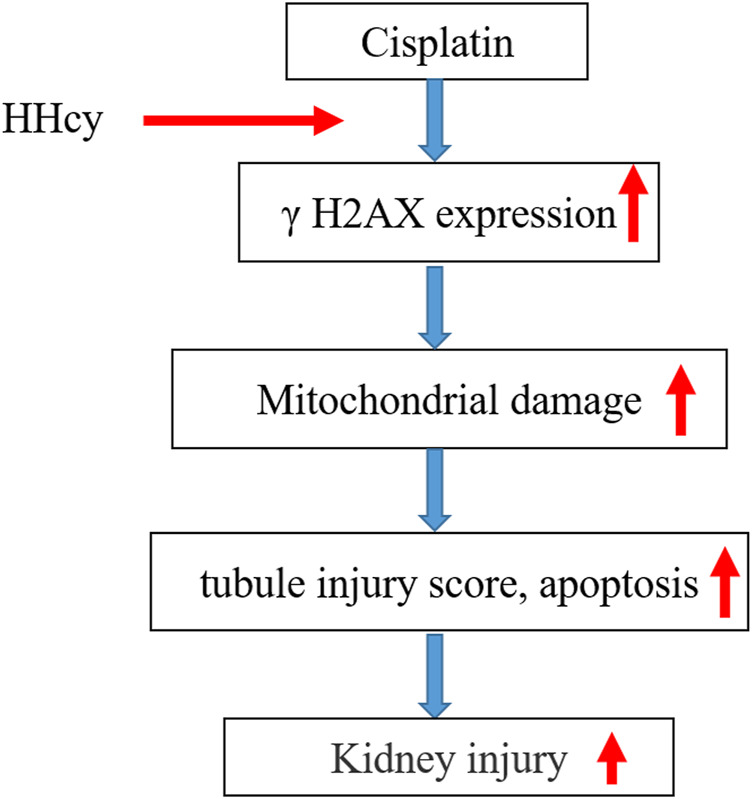
Effect of HHcy on cisplatin-induced renal injury. Elevated plasma homocysteine levels might aggravate DNA damage; this activates mitochondrial damage signaling pathways that further promote kidney injury.

Moreover, in this study, the apoptosis of NRK-52 cells in each group was detected by flow cytometry and western blot respectively. As shown in Figures 5H,I, apoptosis of Hcy + MdiVI-1 cells was significantly reduced compared with the Hcy group. Consistent with flow cytometry results, western blot results showed that the protein expressions of caspase 3 and caspase 12 in NRK-52 cells were significantly decreased after mdivi-1 intervention, compared with the Hcy group (Figures 5J–L). Collectively, these data suggest that HHcy might promote cisplatin-induced AKI by mediating DNA damage and activating the mitochondrial injury signaling pathway (Figure 6).

## Discussion

Owing to huge differences in medical conditions, the mortality rate as a result of AKI in some developing and less well-developed countries is still high. Globally, AKI is not yet effectively prevented. AKI is associated with an increased risk of mortality, cardiovascular events, and progression to chronic kidney disease. Diabetes, aging, and heart failure are strong risk factors for AKI (Levey and James, 2017; Vanmassenhove et al., 2017; Küllmar and Meersch, 2019). Currently, there are no effective drugs for the treatment of AKI. According to Heung and Yessayan (2017), 5% of patients in intensive care with AKI need to start renal replacement therapy, which aggravates the economic and medical pressure on the patients’ families. In response to the “0 by 25” plan of the International Society of Nephrology, the occurrence and deterioration of AKI should be effectively controlled, and the assessment of important risk factors plays an important role in the prevention of AKI.

HHcy has become an independent risk factor for many diseases, including CKD (Hannibal and Blom, 2017; Kong et al., 2017; Peng et al., 2018; Katsimardou et al., 2019). This study found that HHcy plays a functional role in the pathogenesis of cisplatin-induced AKI. Cisplatin-induced AKI has become an ideal rodent model and has been widely used in animal studies (Sharp et al., 2018). In this study, a model of HHcy in mice was established using a high-methionine diet (Hcy level, 49.56 ± 14.08 µM), and a model of AKI was established using a single intraperitoneal injection of 24 mg/kg cisplatin in mice using the short-term high-dose method. We found that, under physiological conditions, HHcy did not affect renal function or renal structure in mice. Interestingly, however, after 1 or 3 days of cisplatin injection, HHcy significantly exacerbated kidney damage in mice. In addition, we detected the expression of DNA damage markers (γ H2AX) in renal tubules. We found that HHcy significantly increased the positive expression of γ H2AX in renal tubular cells in cisplatin-induced AKI. At the same time, our *in vitro* experiments also showed that high concentrations of Hcy could aggravate DNA damage in NRK-52E cells. There is increasing evidence that DNA damage can activate or exacerbate mitochondrial damage pathways. Therefore, we speculate that the mitochondrial injury signaling pathway might play an important role in HHcy in exacerbating AKI.

Renal tubular epithelial cells are among the most oxygen-consuming cells in renal tissue and therefore contain a large number of mitochondria. The mitochondrial injury pathway is one of the main pathways leading to apoptosis of renal tubular epithelial cells. Changes in mitochondrial structure (mitochondrial swelling, mitochondrial crest destruction, and fragmentation) and dysfunction (low mtDNA copy number and reduced ATP synthesis) can be observed in various factor-induced models of AKI (Lisowski et al., 2018; Jiang et al., 2019). A large number of studies have shown that HHcy can aggravate DNA damage in brain tissues, induce mitochondrial damage and mitochondria-mediated apoptosis, and aggravate the course of neurodegenerative diseases (Kumar et al., 2018; Wyse et al., 2019). However, there are few studies on whether HHcy promotes the development of AKI through mitochondrial damage. In this study, we first detected the expressions of ATP β and Cyt C in kidney tissues using western blotting. We found that HHcy slightly decreased renal ATP β expression after cisplatin treatment, but that this result was not statistically significant. This may be due to cell apoptosis when intracellular ATP is abundant, a deficiency in ATP leads to necrosis. However, the ADP/ATP ratio is an important factor for energy consumption and balance (Metallo and Vander Heiden, 2013). We found that HHcy significantly increased ADP/ATP ratio after cisplatin injection. Notably, HHcy significantly accelerated the release of Cyt C into the cytoplasm and reduced the mtDNA copy number. This suggested that HHcy might aggravate mitochondrial damage (Liu R. et al., 2020). Next, we observed, using electron microscopy, that HHcy aggravated the mitochondrial structural damage induced by cisplatin. Finally, we further confirmed that HHcy could promote mitochondrial damage by stimulating NRK-52E cells with 1 mM homocysteine. These results suggest that HHcy may aggravate mitochondrial damage and then lead to apoptosis of renal tubular cells.

In the model of cisplatin-induced AKI, renal tubular epithelial cells are the main target cells, and mitochondrial damage can promote cell apoptosis (Wang et al., 2020; Zhou et al., 2020). To investigate the role of HHcy in cisplatin-induced renal tubular epithelial cells, we detected the number of TUNEL-positive cells in renal tubules using TUNEL detection and the expression of caspase-related apoptotic proteins (cleaved caspase 3 and caspase 12) using western blotting. The viability and apoptosis of renal tubular epithelial cells was evaluated. Our results showed that, 1 or 3 days after intraperitoneal injection of cisplatin, renal TUNEL-positive cell number and expression of cleaved caspase 3 and caspase 12 were significantly increased in mice with HHcy. Compared with *in vivo* inhibition, we found that stimulation of NRK-52 with 1 mM homocysteine significantly increased apoptosis. As Mdivi-1 is an inhibitor of mitochondrial division commonly used *in vivo* and *in vitro* experiments, mainly inhibiting the expression of DRP1, it has been reported that Mdivi-1 can reduce apoptosis of renal tubular epithelial cells caused by cisplatin, ischemia-reperfusion, and sepsis by reducing mitochondrial injury (Li et al., 2018; Liu X. et al., 2020). Interestingly, coculture with Mdivi-1 significantly alleviated Hcy-induced mitochondrial damage and apoptosis. These results suggest that increased Hcy levels will accelerate the apoptosis of renal tubular epithelial cells and promote the development of AKI in mice.

In conclusion, we demonstrated that HHcy might promote the development of AKI by aggravating the apoptosis of renal tubular epithelial cells through the mitochondrial injury pathway (Figure 6). These results suggest that reducing Hcy levels or inhibiting mitochondrial damage may be a potential therapeutic strategy for treating AKI. However, the study only observed that Mdivi-1 could improve HHcy induced mitochondrial damage *in vitro*, which was not combined with *in vivo* experiments. Further studies are warranted to elucidate the role of Mdivi-1 in the HHcy-aggravated AKI mouse model.

## Data Availability

The raw data supporting the conclusions of this article will be made available by the authors, without undue reservation.
